# Challenges and lessons for measuring soil metrics in household surveys

**DOI:** 10.1016/j.geoderma.2020.114500

**Published:** 2020-10-01

**Authors:** Frédéric Kosmowski, Ayale Abebe, Daglar Ozkan

**Affiliations:** a CGIAR Standing Panel on Impact Assessment, Addis Ababa, Ethiopia; b National Soil Testing Center, Addis Ababa, Ethiopia; c Paris School of Economics, Paris, France

**Keywords:** Survey experiment, Soil texture, Soil organic content, Soil acidity, Miniaturized spectrometer

## Abstract

While the importance of soils in agriculture cannot be overlooked, plot level soil data remain scarce in the current data landscape. Large-scale household surveys efforts are increasing in low-income countries and assessing the accuracy, scalability and cost-effectiveness of available methods is crucial. Here, we firstly explore soil data requirements for a set of objectives that include identifying a soil constraint, improving recommendation domain studies and capturing soil metrics as covariates, or as outcomes. We then expose the lessons learned from a methodological experiment in rural Ethiopia, where different approaches – farmer’s self-elicitation and miniaturized spectrometers – are compared against laboratory benchmarks for a set of soil parameters: soil texture, soil pH and soil organic C. With the exception of soil particle sizes, we find that soil parameters captured through farmer’s elicitation do not converge with objective metrics. Miniaturized spectrometers can provide reasonably accurate data for the identification of soil constraints – soil acidity, low organic C or sandy soils. Approximate quantitative predictions can also be delivered for soil pH (R^2^ = 0.72) and organic C (R^2^ = 0.60). The additional costs of plot sampling and analysis are in the range of $19–$23 per sample, with the additional percentage of plots with correct data equivalent to 10% for the identification of sandy soils, 75% for low organic C and 89% of acidic soils.

## Highlights

• The accuracy of farmer’s elicitation and miniaturized spectrometers is assessed in rural Ethiopia.
• Farmer’s elicitation do not converge with objective metrics.
• Miniaturized spectrometers provide accurate data for the identification of soil constraints.
• Miniaturized spectrometers provide approximate quantitative predictions.

## 1 Introduction

The importance of soils in agriculture can hardly be overlooked. Several challenges, including agricultural productivity, food secury, sustainability and climate change cannot be understood and subsequently addressed without accurate soil data. Soil properties are also of particular interest for a wide range of research agendas, including soil erosion, conservation agriculture or natural resource management (Vanlauwe and Giller, 2006). An important number of initiatives – such as Alliance for a Green Revolution in Africa (AGRA), USAID Feed the Future, CIMMYT Sustainable Intensification or IITA Natural Resource Management – have focused on interventions to evaluate and reduce major soil threats in developing countries. These initiatives have been accompanied by a wider interest in producing large scale geospatial datasets (Sanchez, 2009).

The importance of soil in agriculture contrasts with the scarcity of available data, a situation referred by some authors as the “soil data crisis” (McBratney et al., 2006). The path to soil data collection is indeed plagued by challenges. It is admitted that large differences in soil properties exist within farming communities as well as within farms (Walker et al., 1995). Soil nutrient supplies, fertilizer efficiency, and productivity vary widely across small distances: soil heterogeneity at different scales is a major constraint for collecting accurate information. In agricultural surveys, this constraint weights on the costs involved regarding the time-consuming nature of collecting samples representative at the plot level, as well as expensive laboratory analysis. Today, several efforts are devoted to collecting large scale agricultural surveys, from which soil data would be of great additional value. Looking beyond, extensive databases could also allow recommendations based on machine learning methods, which typically need large amount of training data to deliver accurate predictions.

Biophysical studies that use a limited number of measurements can rely on laboratory measurements (Zeleke et al., 2004, Kirubel and Tesfahunegn, 2011). This is not the case for agricultural surveys, where estimates of soil parameters typically rely on farmer’s estimates. Despite soil parameters been extensively used in the economic literature, studies usually fail to account for accurate measures of soil quality when estimating the adoption or impact of agricultural innovations (Duflo et al., 2008, de Janvry et al., 2017).

Since the 2000s, near-infrared spectral methods have been proposed, based on laboratory measurements as well as remote sensing. Near-infrared spectra contain relevant information about soil parameters and an entire strand of the literature have demonstrated their reliability (Cecillon et al., 2008). Several authors have reported accurate NIR predictions for soil organic C (Mouazen et al., 2007; Terhoeven-Urselmans et al., 2008) and soil pH (Shepherd and Walsh, 2002). Wenjun et al. (2014) test the accuracy of visible and near-infrared spectroscopy (350–2500 nm) on soil organic C content. The authors found that in situ spectra had smaller reflectance values than laboratory-based spectra, a fact attributed to soil moisture and ambient light. Authors obtained reasonably accurate predictions (R^2^ = 0.7). Investigating suitable methods for soil organic C in West Africa, McCarty et al. (2010) also found that reflectance spectroscopy performed well.

Recent evidence has delivered conflicting results on the accuracy of farmer’s estimates regarding soil quality. Some studies have undermined the idea that farmer’s self-elicitation correlates with objective measures of soil quality. Studies investigating data collected in three East African countries have shown a weak association, suggesting that farmers use soil type (Berazneva et al., 2018) or soil color and texture as indicators of quality (Gourlay et al., 2017). In the context of Ghana, Dawoe et al. (2012) deliver a more complex picture of the accuracy of farmer’s self-elicitation. While farmer’s assessment are also deemed purely qualitative, there is congruence between objective assessments relying on N, P, K, organic C and pH.

### 1.1 The case for objective measures of soil parameters

In this article, we focus on soil texture, soil pH and soil organic content. This section justifies why these parameters are important data requirements for agricultural surveys.

Soil texture is a permanent characteristic of the soil that affects root growth and overall plant vigor. The relative percentages of sand, silt, and clay are what give soil its texture (USDA, 2019). Soil texture determines the aggregate stability of the soil as well as its compaction and infiltration rate. Sand particles are coarser (2 to 0.05 mm) than silt (0.05 to 0.002 mm) and clay particles (<002 mm). Importantly, water intake and water storage in the soil are primarily controlled by the soil texture and the soil organic C. Finally, soil texture determines the ease of the tillage operations.

Soil acidification is a natural process that can be accelerated by farming practices such as irrigation, fertilization and the removal of crop residues from farmland. Soil acidity affects yields by controlling plant nutrient availability and toxicity. Once acidified, soils become more prone to nutrient loss as well as crops’ absorption of heavy metals (Bolan et al., 2003). Acidic soils can also hinder the profitability of fertilizer, as shown by Burke et al. (2016) in the context of Zambia. This situation has become a concern in several countries. In southern China, 65 percent of agricultural soils have become severely acidified (Guo et al., 2010). In Ethiopia, 80% of Nitisols are severely affected by acidity (Amede et al., 2019).

Soil organic C serves as a reservoir of nutrients and water in the soil and is generally considered as a good proxy of soil quality (Doran and Parkin, 1996, Marenya and Barrett, 2009; Burke et al., 2019). The term refers to any living or dead organism that contains carbon compounds manure, plant material, roots, or living microbial biomass. Influenced by climate, soil type and management practices, soil organic carbon increases or decreases only slowly, moving towards an equilibrium value.

Among these four soil components, it is important to reflect on how objective soil measures should be collected. These measurements could indeed serve different data requirements:

a) the soil data value could indicate a constraint for agriculture, whose identification is important. This is the case for soil texture, as a sandy soil will have low water and nutrient holding capacity. Identically, the identification of acidic soils is of particular relevance in several countries, including Malawi (World Bank, 2018) and Ethiopia (Amede et al., 2019). Finally, low organic C is a major constraint over sub-Saharan Africa. These three metrics could provide a basis for systematic targeting of inputs or technologies.
b) the soil data can be used to develop or further improve recommendation domains. A large area of research has been devoted to the design of recommendation domains (Tesfaye et al., 2015, Gaisberger et al., 2017). Using available sources of data and observed relationships with the technology of interest, these systems predict suitable physical and environmental conditions where an innovation is most likely to perform. Indeed, a large range of agricultural innovations rely on soil parameters for their success and soil data are mostly absent from recommendation domains (Jiménez et al., 2016).
c) the soil data is used as a covariate in regression settings. Several studies make use of soil parameters as covariates. Largely used proxies include soil fertility (Amsalu and de Graaff, 2006, Kato et al., 2009; Jaleta et al., 2013; Kassie et al., 2012, Kondylis et al., 2015) and soil color (Kato et al., 2009, Verkaart et al., 2019). Depending on study objectives, it can be argued that farmer’s estimated fertility is more relevant than true fertility. In that case, the soil metric thus captures a prediction of the underlying value given the farmer’s information set, which includes signals of the true value (Hyslop and Imbens, 2001). Notwithstanding, concerns exist over Non-Classical Measurement Errors (NCME). Noisy measurements of soil parameters can potentially lead to such a situation where statistical noise is not normally distributed (Abay et al., 2019) and analyses are biased.
d) the soil data is the target of an intervention and is expected to change in response to an action, typically a farm management practice. While permanent physical soil properties (for instance, bulk density) are unlikely to be the subject of an intervention, metrics able to capture the evolution of organic C or soil pH would be of strong interest. Unlike covariates, where capturing farmer’s perception rather than the true value of the variable may be relevant, soil metrics as an outcome may not be accurately captured by subjective measurements.

These different objectives call for different metrics and precision levels. When identifying a soil constraint (a), the relevant metric is a binary variable (ex: acidic soil *vs* non acidic soil). When used as covariates (c), soil parameters could in theory accommodate all types of measurement, although the more precision the better. Other data uses (b, d) often require a variable that spans across the entire range of values of the parameters. While a numeric variable would represent the highest level of precision, in practice it may be possible to rely on an ordinal measure.

The general objective of this paper is to advance the discussion on survey methods and provide practitioners guidance when conducting survey work and analysis. The strategy is *i*) to document the extent of measurement errors associated with farmer’s elicitation; *ii*) to explore the accuracy of miniaturized spectrometers, as a potentially field-based, scalable device and *iii*) provide a ranking of these methods in terms of cost and accuracy for future large scale agricultural surveys.

## 2 Data and methods

### 2.1 Data collection

Survey data was collected in January 2018 in seven enumeration areas in Ethiopia (Table 1). These areas were selected to obtain a fairly diverse representation of soil types and fertility status (EthioSIS, 2016). In each enumeration area, households were listed, and a random sample of 40 households was interviewed. The questionnaire comprised six sections and is available in Appendix A. In each household, a random plot was selected, and farmer’s elicitation was collected for this specific plot, for the set of soil parameters studied. Enumerators then visited the plot for soil sampling. Data were collected with tablets equipped with Survey Solutions. A total of N = 284 households-plots was surveyed.

**Table 1:** Surveyed areas.

Zone	Site (District)	Latitude	Longitude	Mean altitude (in m)
North Shewa	Majana Wedera	9.890489	39.48453	2736
Gurage	Sodo Woreda	8.168632	38.64645	1912
Gurage	Muhir Na Aklil	8.133279	38.20867	3248
Gurage	Ezha	8.179497	37.92722	2019
Oromia	Dawa Harewa	10.72069	39.88603	1416
North Wollo	Meket	11.814	39.14041	3274
North Wollo	Wadla	11.65492	39.03575	3026

Soil sampling was performed using a star design protocol. First, enumerators identified the plot center. With the help of compass and GPS, enumerators had to collect eight sample points, each at 45 degrees, starting from the Northern direction (Appendix A, section 6). From the plot center, samples in direction of the four cardinal points were collected at half distance from the plot boundary, while samples between cardinal points (for instance, North-West) were collected at three quarters of the distance between the plot center and the plot boundary. At each sampling point, three topsoil samples (0–20 cm) were collected with a tubular soil sampler and mixed in a bucket to make a composite. The composite sample was placed in a zipped plastic bag and barcoded. While time consuming (average protocol duration was 55 min), this protocol insured the representativeness of soil parameters at the plot level.

Soil samples were air dried and ground with mortar and pestle to pass through a 2 mm sieve and subjected to analysis at the Ethiopian National Soil Testing Center. Soil particle size was determined using the Bouyoucos hydrometer Method (Gee and Bauder, 1986). Soil organic carbon (OC) was determined by the Walkley-Black wet oxidation method (Walkley and Black, 1934) while soil pH was measured in the supernatant suspension of a 1:2.5 soil to water mixture using a pH meter (Van Reeuwijk, 2002). Due to a lack of available material, soil organic C was determined on n = 242 samples. Other soil parameters were assessed on the entire sample (n = 284).

In this study, the benchmark values from each soil parameters are compared to two alternative methods: farmer’s elicitations and miniaturized spectrometers (Table 2).

**Table 2:** Overview of methods used for estimating soil characteristics.

	Benchmark	Alternative methods	
		Farmer's elicitation	Miniaturized NIR spectrometers
Soil texture	Chemistry: Hydrometer	Silt/Sand/Clay and particles size	Yes
pH	Chemistry: Potentiometry	Acidic or not	Yes
Soil Organic C	Chemistry: Walkley-Black method	Good/Fair/Poor and 10-level scale	Yes

### 2.2 Spectral data analysis

Recent years have seen the commercialization of miniaturized sensors based on the NIR spectroscopy technique (Rateni et al., 2017, Kosmowski and Worku, 2018). Miniaturized NIR spectrometers require minimal equipment while also offering the advantages of portability. Two miniaturized devices are used in this study. First, the Consumer Physics SCIO, delivered as a $1,000 developer kit at the time of writing. The device’s full wavelength coverage is 740–1070 nm. Second, the Tellspec, equipped with the DLP® NIRscan™ Nano had a price of $2,000 at the time of writing. Its full wavelength coverage is 900–1700 nm. When samples are scanned, a signature is generated through vibrational stretching and bending of structural groups of atoms (Genot et al., 2011). Spectral signatures are defined as their absorbance (log 1/R) as a function of wavelengths.

Pre-processing methods are helpful in eliminating noise generated by spectral data. Raw spectral data were processed using a combination of scatter corrections. In our evaluation, we then compare the performance of several algorithms: partial least squares discriminant analysis, random forest, neural networks, support vector machine and logitBoost for classification; linear regression, partial least squares discriminant analysis, ridge regression, enet regression, random forest, support vector machine and neural networks for regression. A description of these algorithms can be found in Gareth et al. (2013).

In the absence of a large test set to estimate the error rates, resampling methods appear preferable. It is known that using *k*-fold cross-validation, bias and variance properties are acceptable and reasonable predictions can be produced (Kuhn and Johnson, 2013). In order to assess the accuracy of these algorithms in predicting soil parameters, we used 10-fold cross-validation. The error is the difference between the predicted and observed values of performance. Oversampling methods were used in order to address class imbalance (for sandy soils and acidic soils binary models). Algorithm parameters were optimized randomly during cross-validation.

The best performing model was selected using sensitivity, or percentage of actual positives correctly classified (for binary classifications) and RMSE (for regressions). We used R 3.6.0 (R Core Team, 2019), along with the packages prospectr (Stevens and Ramirez-Lopez, 2013) and caret (Kuhn, 2008). For each soil parameter, the raw spectra and 13 preprocessing techniques were used to train and validate each algorithm. Along with the datasets in Appendix B, a reproducible script is available in Appendix C.

## 3 Results

### 3.1 Soil characteristics

**Fig. 1:**
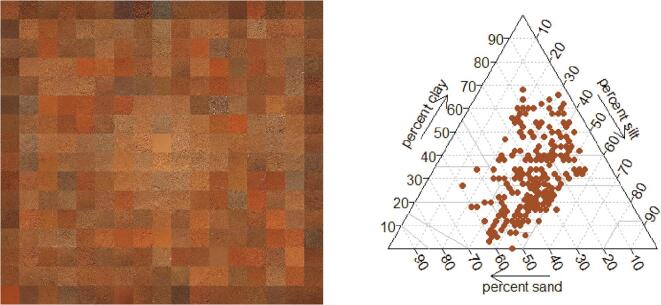
Visual representation of collected soil samples (a) and soil textural class distribution in the survey area (b).

Mean Organic C concentration from the sample ranged from 1.2 to 3.4%. These levels are in line of those of previously reported studies in Ethiopia (Corral-Nuñez et al., 2014). The soil pH was found to range from acidic to alkaline (5.1 to 7.9) with a majority of samples having a neutral pH. Munsell values are predominantly composed of light and moderate intensities of brown, with color values ranging from 5.2 to 6.9 (Fig. 1a). Ezha and Wadla areas stand out of the surveyed areas, having darker colors than elsewhere. The range in textural class distribution of the soils is shown in Fig. 1b. The sample is fairly well distributed between the different soil classes with loam soils, clay loam and clay soils being dominant in the surveyed areas.

### 3.2 Relationships between parameters

**Fig. 2:**
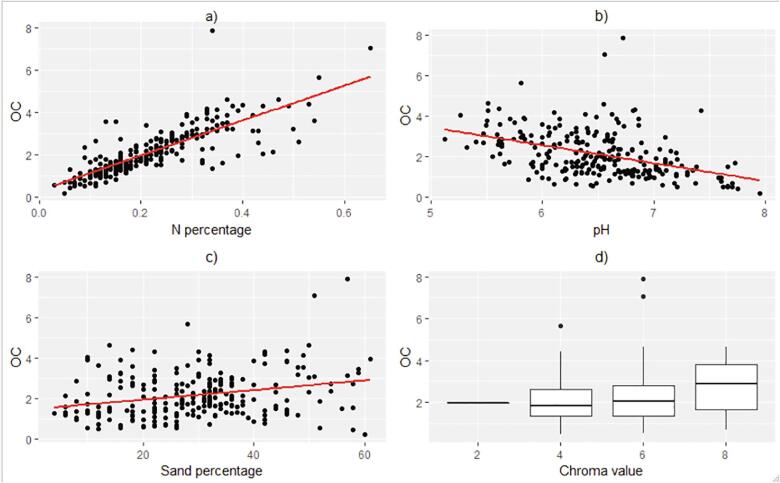
Relationship between organic C and N percentage (a), pH (b), sand percentage (c) and chroma value (d).

It is agreed that a unique relationship exists for different soil landscapes, due to differences in soil composition and mineralogy (Konen et al., 2003). Associations between soil organic C and several other soil parameters have however been reported in the literature (Wills et al., 2007). In Fig. 2, we examine these relationships. Total nitrogen (TN), a critical element for soil fertility appear to highly correlate with soil organic C. Other parameters do not appear to be associated with organic C in the surveyed area. Soil pH shows a weak but negative relationship with organic C. Chroma values – how weak or strong a color appears – are believed to be determined by humus (dark brown color) and the type of iron compound. A clear relationship would allow the determination of organic C content based on soil color solely. However, it is clear from Fig. 2.d that chroma values are unrelated to organic matter levels.

### 3.3 Comparison between laboratory benchmarks and alternative methods

In this section, we evaluate two sources of soil data: farmer’s elicitation and miniaturized spectrometers against a laboratory benchmark.

#### 3.3.1 Soil texture

Soil texture has important implications for water and nutrient uptake. It is known that lower the sand percentage, the lower water holding capacities. Soils dominated by sand retain less water and are thus more prone to the effect of drought. Given these, the identification of sandy soils should be a data priority. A sandy soil is defined as a parcel whose texture is classified as sand, loamy sand or sandy loam soils, following the USDA soil classification (USDA, 2019). The percentage of sand particles ranges between 20% and 60% in the surveyed area.

**Fig. 3:**
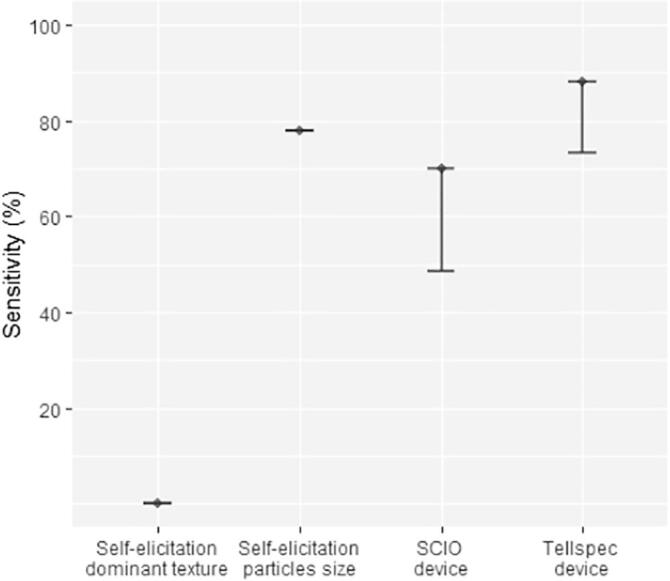
Sensitivity (% of positives correctly classified) of alternative methods of data collection for measuring sandy soils (binary classifier). For SCIO and Tellspec devices, the lower bound indicates one standard deviation from the selected algorithm.

**Fig. 4:**
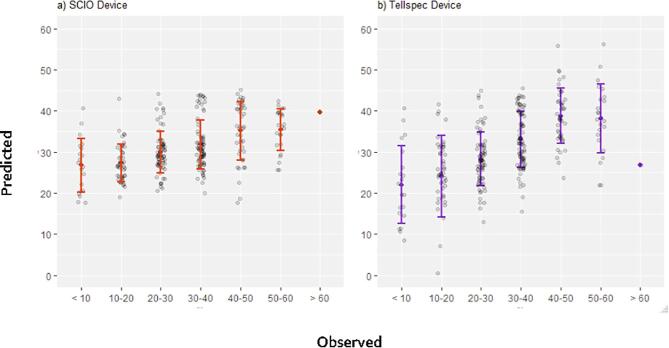
Plots of observed *vs*. predicted values of sand percentage for the best performing algorithms using spectral data from SCIO (a) and Tellspec devices (b). For all models, a 10-fold cross validation was performed. Error bars indicate one standard deviation of the mean.

The data collected on soil texture included two questions capturing farmer’s self-elicitation. The first question asked, “What is the dominant soil texture on this parcel?” with clay, silt and sand as possible answers. The second question, “What is the size of soil particles on this parcel?” was captured on a Likert scale (Very fine; fine; between coarse and fine; coarse and very coarse). Results are presented in Fig. 3. Regarding the binary classifier, self-elicitation based on soil dominant texture largely miss out sandy soils. Asking farmers about particle sizes shows better promises, with 78% of sandy soils identified as “coarse.” However, in terms of overall accuracy, the question still performs below standards (43% accuracy). The Tellspec identifies 88% of soils as sandy (and 12% as not sandy), and these classifications are correct for 77% of the samples. The spectrometer devices could only deliver poor quantitative predictions, with a R^2^ of 0.21 and 0.33 for the SCIO and Tellspec devices respectively (See Fig. 4).

#### 3.3.2 Soil organic C

Soil organic matter has been the subject of extensive research and is a favored proxy for soil fertility diagnostics (Doran and Parkin, 1996, Marenya and Barrett, 2009; Genot et al., 2011). A commonly used question in agricultural surveys is “What is the soil quality of this parcel?” with good, fair and poor as possible answers. Additionally, we included the question: “On a scale from one to ten, how would you rate the soil quality of this parcel?”. A large majority of farmers (60%) classified their parcel soil fertility as fair. Sixteen percent judged it as good and twenty-four percent as poor. Similarly, the 10-levels assessment of soil fertility approximates a normal distribution, with a mean of 5.3 and a standard deviation of 2.

**Fig. 5:**
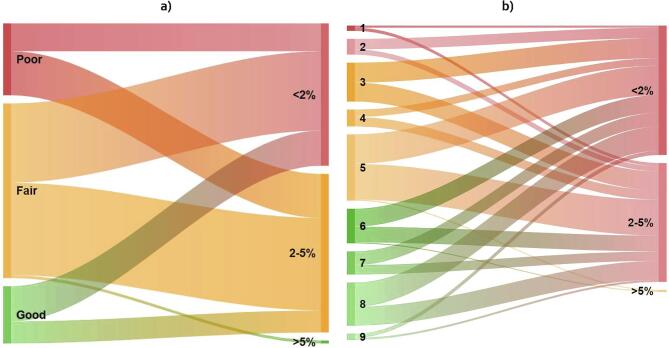
Sankey diagram capturing the relationship between farmer’s elicitation on parcel fertility using ordinal categories (a) or a ten levels scale (b). The right side of each diagram shows organic C content from laboratory analysis.

Results, presented in Fig. 5, demonstrate the absence of correlation of organic C with farmer’s perception on soil fertility. No consistent pattern is found and soils with less than 2% organic C (considered as poor soils) are almost equally distributed between the fertility levels. When asked about the characteristics of a fertile soil, farmers primarily cite yields, followed by soil workability, soil type, darker soil and soils with high water holding capacities.

**Fig. 6:**
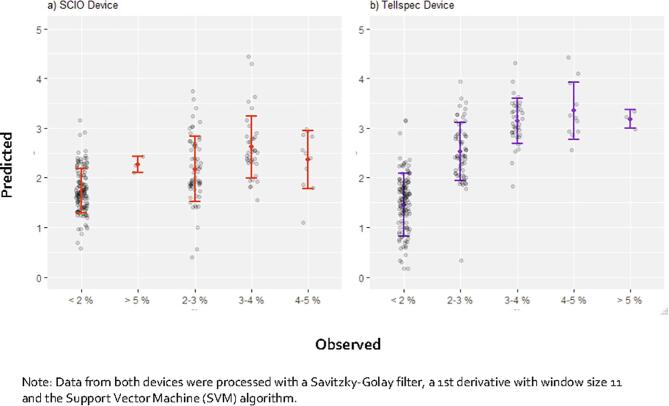
Plots of observed *vs*. predicted values of organic C for of the best performing algorithms using spectral data from SCIO (a) and Tellspec devices (b). For all models, a 10-fold cross validation was performed. Error bars indicate one standard deviation of the mean.

We then explore the accuracy of miniaturized spectrometers, as shown in Table 4. The binary classifier seeks to identify infertile soils, defined as parcels with organic carbon lower than 2%. Both devices achieved acceptable rates of identification, with 85% and 94% of low organic C content soils identified by the SCIO and the Tellspec respectively. Regarding quantitative predictors, the second device only (Tellspec) yield approximate predictions (R^2^ = 0.60), with a clear positive relationship (Fig. 6).

**Table 4:** Sensitivity (% of positives correctly classified) and accuracy (% correctly classified) of alternative methods of data collection for organic C.

	% Sensitivity	% Accuracy
Self-elicitation	19	11
SCIO device	85	79
Tellspec device	94	87

*Note*: SCIO raw data was processed with the Support Vector Machine (SVM) algorithm; Tellspec data was processed with a Savitzky-Golay filter, a 2nd derivative with window size 5 and the Random Forest (RF) algorithm.

Given the encouraging results provided by the Tellspec, we further explore wavelengths associated with different levels of soil organic C in Appendix D. Absorption features near the 1430–1436 nm and the 1649–1668 nm regions appear highly associated with Organic C.

#### 3.3.3 Soil pH

**Fig. 7:**
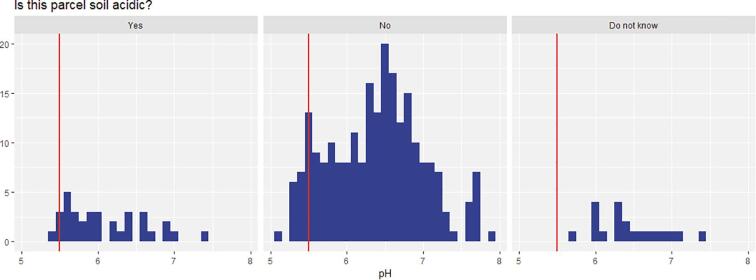
Histogram of farmer’s elicitation on plot acidity compared with laboratory measurements. A value lower than 5.5 indicates an acidic soil (red line). (For interpretation of the references to color in this figure legend, the reader is referred to the web version of this article.)

Our last soil parameter to consider is soil acidity. Defined as a soil with a pH lower than 5.5, acidity is a serious threat in sub-Saharan Africa. Fig. 7 displays farmer’s elicitation against the laboratory benchmark. Staggeringly few (8%) farmers who have an acidic soil parcel were aware of it. This result has important implications as one can hardly tackle an agricultural constraint that is unknown. Using farmer’s elicitation, most positive answers to the question “Is this parcel soil acidic?” happen to be false positives.

**Fig. 8:**
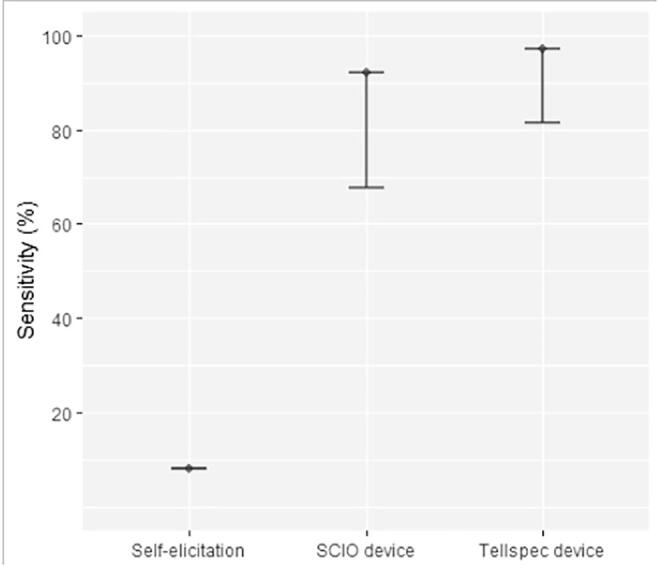
Sensitivity (% of positives correctly classified) of alternative methods of data collection for measuring acidic soils (binary classifier). For SCIO and Tellspec devices, the lower bound indicates one standard deviation from the selected algorithm.

**Fig. 9:**
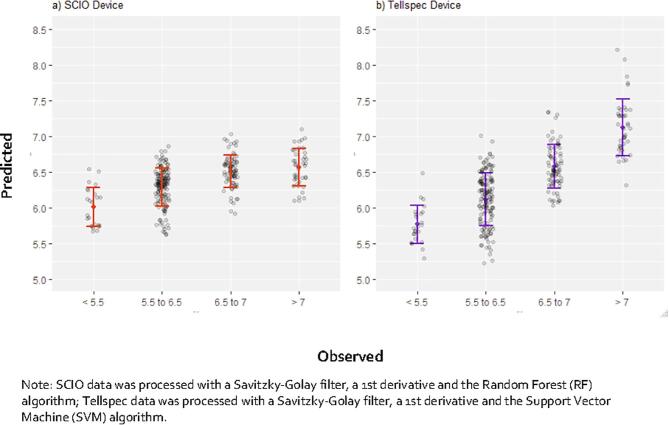
Plots of observed *vs*. predicted of the best performing algorithms using spectral data from SCIO (a) and Tellspec devices (b). For all models, a 10-fold cross validation was performed. Error bars indicate one standard deviation of the mean.

Once again, miniaturized spectrometers appear to better capture soil acidity than farmer’s elicitation (Fig. 8). The Tellspec device classifies 97% of acidic soil, with an overall accuracy of 84%. The SCIO device provides sensitivity above 90% for the identification of acidic soils, but fails to capture soil pH on a numeric scale (Fig. 9a). Results of the regression models indicate and R^2^ of 0.72 for the Tellspec device. This can be confirmed visually from Fig. 9b, with more data points clustered around the 45 degrees dashed lines.

All soil parameters considered, pre-processing methods brought an average diminution of 7% RMSE, compared to raw data. The gain in accuracy from using the Tellspec over the SCIO is 30% for soil organic C and 36% for soil pH. It is noticeable that for most soil parameters, the wider wavelength range provided by the Tellspec device (900–1700 nm, compared to 740–1070 nm for the SCIO) provides a significant advantage in terms of accuracy.

### 3.4 Costs associated with soil sampling

In this section, we seek to estimate the additional cost of collecting and analyzing soil samples in a household survey. The cost of objective methods equals the additional time spent by enumerators collecting plot-level samples with the method-specific cost of analysis. In our calculations, we included two laboratory costs estimates: the price per sample at the Ethiopian National Soil Testing Center; and the price per sample at the Soil-Plant Spectral Diagnostics Laboratory (World Agroforestry Center) in Kenya. A major institution in soil analysis, the latter has provided support to several soil research institutes in sub-Saharan Africa.

Mean plot size in the surveyed area was 4240 square meters. On average, randomly selected plots were found at 18 min walking distance from the household and soil sampling duration was 55 min. Cost estimates are presented in Table 6. More than 80% of the additional cost is related to soil sampling. Given the low accuracy of self-elicitation methods, our interest here is to compare the use of miniaturized spectrometers with conventional laboratory analysis. With binary classifiers, the additional cost of using miniaturized spectrometers are about $19 per sample, with additional percentage of plots with correct data equivalent to 10% for sandy soils, 75% for low organic C and 89% for acidic soils identification. Laboratory analysis costs range from $19 to $22 depending on the parameter studied; while the additional percentage of plots with correct metrics range from 22% to 92%. In comparison to laboratory analysis, miniaturized spectrometers provide slightly less accurate results at a reduced cost. When precise soil metrics needs to be collected on a numeric scale, the relative superiority of laboratory measurements is clearly established.

**Table 6:** Cost associated with the inclusion of soil sampling for measuring soil parameters (2018 USD). Farmer’s elicitation is used as benchmark.

	Sandy soils	Low organic C	Acidic pH
Number of plots	284	242	284

Miniaturized spectrometers			
Additional number of plots that have correct response	30	181	252
Additional % of plots that have correct response	10	75	89
Additional cost (in USD)	18.8	18.8	18.8

**Laboratory analysis/digital camera**			
Additional number of plots that have correct response	63	196	261
Additional % of plots that have correct response	22	81	92
Additional cost for wet chemistry, NSTC Ethiopia (in USD)	20	20	19
Additional cost for wet chemistry (10%) and NIR, WAC Kenya (in USD)	23	22	22

## 4 Discussion

Our research makes two contributions. First, it provides evidence on the accuracy of data collected for a set of soil parameters that were so far absent from the literature: soil pH and soil texture. In accordance with other studies focusing on soil organic C (Gourlay et al., 2017, Berazneva et al., 2018), we find that soil dominant texture and soil pH captured through farmer’s elicitation do not convergence with objective metrics. All things considered, caution should be observed when soil metrics are used in a study and objective metrics should be favored in future survey designs.

Soil texture analysis can certainly benefit from the recent advances in deep learning, a field that has achieved tremendous progress in the domain of image pattern recognition (Waldrop, 2019). Assuming a large enough training set of images is available, soil granularity could be potentially learned and subsequently predicted^1^.

Furthermore, we have demonstrated the promising tool that miniaturized spectrometers could represent for future survey designs. Miniaturized spectrometers can provide reasonably accurate data for the identification of soil constraints as well as approximate quantitative predictions for soil pH and organic C. The tested devices could potentially be applied on-field: after soil sampling, the workflow could consist in establishing a mini-lab where grinding, drying and scanning would be performed, ideally moving with the survey team. In-situ spectra, due to ambient light and soil moisture content, were found to decrease the accuracy of predictions in Wenjun et al. (2014). For miniaturized spectrometers to be used in-field, removing the effects of light and soil moisture appears crucial. How to overcome this issue is a future avenue for research. Finally, it is worth mentioning that newly available devices, with larger spectral ranges (1350–250 nm) could provide more accurate results (Neospectra, 2019).

A caveat from this study concerns its external validity. Since efforts were directed at plot-level representativeness in order to obtain a valid benchmark, our sample is representative of a few enumeration areas only.

Recognized as an increasing threat in sub-Saharan Africa, soil pH is probably the parameter that would benefit the most from the use of miniaturized spectrometers. Binary classifiers of acidic *vs* non-acidic soil achieved acceptable levels of accuracy. The puzzling results that farmers with acidic soils were unaware of it certainly deserves more scrutiny. In the event of large-scale programs that aim at rehabilitating acidic soils, for instance through lime applications, farmers’ information and knowledge are likely to play a crucial role in taking measures to reduce soil acidity.

## 5 Conclusions

In this article, we have described soil metrics data requirements for some of the most common research objectives. From government-led household surveys to impact assessments of agricultural technologies, there is a need to assess available options, both in terms of accuracy and costs. We did so by presenting results of a methodological experiment in Ethiopia where farmer’s self-elicitation and miniaturized spectrometers were compared against laboratory benchmarks for soil texture, soil pH and soil organic C. Our findings suggest the questionable nature of subjective methods in future survey designs. Our results have demonstrated the suitability of miniaturized NIR spectrometers for studies interested in identifying soil constraints – soil acidity, low organic C or sandy soils; and where approximate quantitative predictions for soil pH and organic C are acceptable. We hope that these insights will be taken over by survey practitioners as well as data analysts. The cost of soil metrics accuracy may seem high and upscaling plot-level sampling might not be affordable for resource-constrained statistical institutes. This new frontier in household survey designs however needs to be crossed for agricultural technology uptake to be well understood, and their potential impact on farmer’s livelihoods to be evaluated.

## Declaration of Competing Interest

The authors declare that they have no known competing financial interests or personal relationships that could have appeared to influence the work reported in this paper.

## Footnotes

1 Our dataset of soil images is available upon request.
