# Supplementary material for: Challenges and lessons for measuring soil metrics in household surveys
Source: Geoderma. 2020 Oct 1;375:114500. doi: 10.1016/j.geoderma.2020.114500 (PMC7386900; doi:10.1016/j.geoderma.2020.114500)
Supplement: Supplementary data 1 [file mmc1.zip › Appendix A. Questionnaire]

This PDF generated by fkosmowski, 1/5/2018 1:22:59 AM
Questionnaire created by tigist, 7/19/2017 6:09:21 AM
Last modified by fkosmowski, 1/5/2018 6:22:51 AM

Not shared with anyone

Sections: 6, Sub-sections: 6,
Questions: 123.
Questions with enabling conditions: 39
Questions with validation conditions:5
Rosters: 2
Variables: 12

Soil Quality Survey (SQS)

SECTION 1: COVER
No sub-sections, No rosters, Questions: 6.

SECTION 2: HOUSEHOLD CHARACTERISTICS
No sub-sections, No rosters, Questions: 11.

SECTION 3: PARCEL DESCRIPTION
Sub-sections: 2, Rosters: 1, Questions: 25, Static texts: 3, Variables: 4.

SECTION 4: FIELD DESCRIPTION
No sub-sections, Rosters: 1, Questions: 29.

SECTION 5: INDIGENOUS SOIL FERTILITY PERCEPTION
No sub-sections, No rosters, Questions: 10, Static texts: 1.

SECTION 6: ENUMERATOR PARCEL VISIT
Sub-sections: 4, No rosters, Questions: 42, Static texts: 37, Variables: 8.

LEGEND

SECTION 1: COVER

1.1. Start time	DATE: CURRENT TIME S1Q1<br>..
1.2. Household ID number	NUMERIC: INTEGER S1Q2<br>_ _ _ _ _ _ _ _ _ _ _ _ _ _ _ _ _ _ _ _
1.4. Interviewer Name	SINGLE-SELECT S1Q4<br>SCOPE: IDENTIFYING<br>01 ○ Enum1<br>02 ○ Enum2<br>03 ○ Enum3<br>04 ○ Enum4<br>05 ○ Enum5<br>06 ○ Enum6
1.5. Enumeration Area	SINGLE-SELECT S1Q5<br>SCOPE: IDENTIFYING<br>01 ○ EA1<br>02 ○ EA2<br>03 ○ EA3<br>04 ○ EA4<br>05 ○ EA5<br>06 ○ EA6<br>07 ○ EA7<br>08 ○ EA8<br>09 ○ EA9<br>10 ○ EA10
1.6. Respondent Name	TEXT S1Q6<br>..
1.7. Place of the interview	SINGLE-SELECT S1Q7<br>01 ○ Household<br>02 ○ Parcel<br>03 ○ Other

SECTION 2: HOUSEHOLD CHARACTERISTICS

2.1. Respondent relation to the household head	SINGLE-SELECT S2Q1<br>01 ○ Household head<br>02 ○ Spouse<br>03 ○ Daughter<br>04 ○ Son<br>05 ○ Other
2.2. Sex of the respondent	SINGLE-SELECT S2Q2<br>01 ○ Male<br>02 ○ Female
2.3. Age of respondent	SINGLE-SELECT S2Q3<br>01 ○ <20 years<br>02 ○ 20-30 years<br>03 ○ 31-40 years<br>04 ○ 41-60 years<br>05 ○ >60 years

2.4. What is the highest level of school you have attended?	SINGLE-SELECT S2Q4<br>01 ○ Adult literacy Program<br>02 ○ Primary<br>03 ○ Secondary<br>04 ○ Technical/vocational<br>05 ○ Higher<br>06 ○ Not Educated<br>07 ○ Don't know
2.5. What is the completed education level of the respondent?<br>I 00 = LESS THAN 1 YEAR COMPLETED IF PRIMARY OR SECONDARY, RE CORD COMPLETED GRADE. IF TECHNICAL/VOCATIONAL OR HIGHER, RECORD YEARS COMPLETED.<br>E S2Q4<6	NUMERIC: INTEGER S2Q5<br>_ _
2.6. What is the household size of the respondent?<br>V1 S2Q5<15<br>M1 House hold size should be less than 15	NUMERIC: INTEGER S2Q6<br>_ _
2.7. How many (working) mobile phones do you own in this household?<br>I THEY SHOULD COUNT ALL MOBILE PHONES OWNED BY ANY OF THE HOUSEHOLD MEMBERS	NUMERIC: INTEGER S2Q7<br>_ _
2.8. Do you belong to a farmer group or agricultural cooperative?<br>I Group of farmers cooperating to obtain agricultural inputs or sell prod uction	SINGLE-SELECT S2Q8<br>01 ○ Yes<br>02 ○ No
2.9. Were you under Extension Program during the current agricultural season?	SINGLE-SELECT S2Q9<br>01 ○ Yes<br>02 ○ No
2.10. How many times have you been visited by a Development Agent (DA) from extension services in the past 12 months?<br>E S2Q9==1	NUMERIC: INTEGER S2Q10<br>_ _
2.11. How many time in the last 6 months did you attend a meeting at the Agricultural extension services?	NUMERIC: INTEGER S2Q11<br>_ _

SECTION 3: PARCEL DESCRIPTION

STATIC TEXT

RANDOM SELECTION OF A PARCEL

A PARCEL OF LESS THAN 1 HOUR WALKING DISTANCE WILL BE RANDOMLY SELECTED

3.1. LIST ALL PARCELS OF LAND OWNED OR RENTED IN (BOTH CULTIVATED AND NON CULTIVATED)	LIST S3Q1<br>..

SECTION 3: PARCEL DESCRIPTION

PARCEL DETAILS

E S3Q1.Count()>0

SECTION 3: PARCEL DESCRIPTION / PARCEL DETAILS

Roster: 3.2. PARCEL

generated by list question S3Q1 S3Q2

3.3. What is the PARCEL ID of %rostertitle%?	NUMERIC: INTEGER S3Q3<br>_ .
3.4. What is the area of %rostertitle% in hectares?	NUMERIC: DECIMAL S3Q4<br>_ .
3.5. How did your household acquire this %rostertitle%?	SINGLE-SELECT S3Q5<br>01 ○ Granted by local leaders<br>02 ○ Inherited<br>03 ○ Rent<br>04 ○ Borrowed for free<br>05 ○ Moved in with out Permission<br>06 ○ Shared Crop in<br>07 ○ Purchased<br>08 ○ Other
3.6. Do you have the right to sell %rostertitle%?	SINGLE-SELECT S3Q6<br>01 ○ Yes<br>02 ○ No
3.7. Does anyone in the household have the right to sell %rostertitle%?<br>E S3Q6==2	SINGLE-SELECT S3Q7<br>01 ○ Yes<br>02 ○ No
3.8. What is the predominant soil type of this %rostertitle%?	SINGLE-SELECT S3Q8<br>01 ○ Leptosol (Ashewama afer)<br>02 ○ Cambisol (Bicha Afer)<br>03 ○ Vertisol (Tikure afer)<br>04 ○ Luvisol (Keye afer)<br>05 ○ Mixed type (Debeleq afer)<br>06 ○ Other type
3.9. How would you name the type of soil on %rostertitle% (in Amharic)?<br>I RECORD AMHARIC WORD USED. DO NOT KNOW IS AN ACCEPTED AN SWER	TEXT S3Q9<br>..
3.10. What is the soil quality of %rostertitle%?	SINGLE-SELECT S3Q10<br>01 ○ Good<br>02 ○ Fair<br>03 ○ Poor
3.11. How many minutes does it take to travel from the homestead to %rostertitle% by walk?	SINGLE-SELECT S3Q11<br>01 ○ Less than 15 Mins<br>02 ○ 15 - 30 Mins<br>03 ○ 30 - 59 Mins<br>04 ○ 1 - 2 Hours<br>05 ○ Over 2 Hours
3.11b. During the last three years, have you planted a legume on %rostertitle%<br>I LEGUMES ARE CHICKPEA, HARICOT BEANS, FADA BEANS, LENTILS	SINGLE-SELECT S3Q11b<br>01 ○ Yes<br>02 ○ No

STATIC TEXT

E S3Q12==0

IF NO ELIGIBLE PARCEL, END THE SURVEY

VARIABLE<br>S3Q2.Count($criteria)	LONG	S3Q12
VARIABLE<br>(long)Math.Floor(Quest.IRnd()*S3Q12.Value)	LONG	S3Q13
VARIABLE<br>S3Q2.Where($criteria).Select(z=>z.@rowcode).ToArray()[S3Q13.Value]	LONG	S3Q14
VARIABLE<br>S3Q1.Where(x=>x.Item1==S3Q14).First().Item2	STRING	S3Q15

STATIC TEXT

CONTINUE THE INTERVIEW WITH PARCEL %S3Q15%

SECTION 3: PARCEL DESCRIPTION

SOIL QUALITY ASSESSMENT

3.16. What is the soil color of %S3Q15%?<br><br>I DO NOT CITE, FIND THE CLOSEST ANSWER	SINGLE-SELECT<br>01 ○ Light Grey (Fezaza Geracha)<br>02 ○ Dark Grey (Tekore yale geracha)<br>03 ○ Black (Tikur)<br>04 ○ Grey-Brown (gerachama buni/ teyem)<br>05 ○ Light Brown (Fezaza buni)<br>06 ○ Moderate Brown (mekakelegna buni)<br>07 ○ Dark Brown (tiqure buni)<br>08 ○ Pale Orange (Fezaza bertekuanema)<br>09 ○ Yellow-Orange (Bicha bertukanama)<br>10 ○ Yellow-Brown (Bicha buni)<br>11 ○ Other (specify)	S3Q16
Other: Precise the color<br><br>I Translate the color in english<br>E S3Q16==11	TEXT<br>..	S3Q16a
3.17. VISUAL-AID-PROTOCOL: In which "block" is the soil color of %S3Q15%?<br><br>I USE THE RELEVANT VISUAL AID PROTOCOL	SINGLE-SELECT<br>01 ○ N<br>02 ○ 5YR<br>03 ○ 10YR	S3Q17
3.18. What is the soil color of %S3Q15%?<br><br>E S3Q17==1	SINGLE-SELECT<br>01 ○ N8<br>02 ○ N6<br>03 ○ N4<br>04 ○ N2<br>05 ○ N1	S3Q18
3.19. What is the soil color of %S3Q15%?<br><br>E S3Q17==2	SINGLE-SELECT<br>01 ○ 5YR 8/2<br>02 ○ 5YR 8/4<br>03 ○ 5YR 6/2<br>04 ○ 5YR 6/4	S3Q19

05 ○ 5YR 6/6
06 ○ 5YR 6/8
07 ○ 5YR 4/2
08 ○ 5YR 4/4
09 ○ 5YR 4/6
10 ○ 5YR 4/8
11 ○ 5YR 2/2
12 ○ 5YR 2/4
13 ○ 5YR 2/6

3.20. What is the soil color of %S3Q15%?

E S3Q17==3

SINGLE-SELECT S3Q20

01 ○ 10YR 8/2
02 ○ 10YR 8/4
03 ○ 10YR 8/6
04 ○ 10YR 8/8
05 ○ 10YR 6/2
06 ○ 10YR 6/4
07 ○ 10YR 6/6
08 ○ 10YR 6/8
09 ○ 10YR 4/2
10 ○ 10YR 4/4
11 ○ 10YR 4/6
12 ○ 10YR 2/2
13 ○ 10YR 2/4

3.21. What is the slope of %S3Q15%?

SINGLE-SELECT S3Q21

01 ○ Flat / nearly flat (Tefetafa/medama)
02 ○ Gentle / moderate slope (Medama mekakelegna daget)
03 ○ Strong slope (Daget)
04 ○ Steep slope (Akebet)

3.22. VISUAL-AID-PROTOCOL: What is the slope of %S3Q15%?

I USE THE RELEVANT VISUAL AID PROTOCOL

SINGLE-SELECT S3Q22

01 ○ Picture 1
02 ○ Picture 2
03 ○ Picture 3
04 ○ Picture 4
05 ○ Picture 5
06 ○ Picture 6
07 ○ Picture 7

3.23. What is the % of rocks on %S3Q15%?

SINGLE-SELECT S3Q23

01 ○ 0%
02 ○ 2%
03 ○ 5%
04 ○ 10%
05 ○ 20%
06 ○ 30%
07 ○ 50% and more

3.24. VISUAL-AID-PROTOCOL: What is the % of rocks on %S3Q15%?

I USE THE RELEVANT VISUAL AID PROTOCOL

SINGLE-SELECT S3Q24

01 ○ Picture 1
02 ○ Picture 2
03 ○ Picture 3
04 ○ Picture 4
05 ○ Picture 5
06 ○ Picture 6
07 ○ Picture 7

3.25. What is the dominant soil texture of %S3Q15% ?

SINGLE-SELECT S3Q25

01 ○ Silt (Betam dekake/ lem afer)
02 ○ Sand (Ashewa Afer)
03 ○ Clay (Shekela Afer)

	04 ○ Do not know
3.26. What is the size of soil particules on %S3Q15%?	SINGLE-SELECT S3Q26<br>01 ○ Very Fine (betam Dekake)<br>02 ○ Fine (Dekake)<br>03 ○ Between coarse and fine (Be shekara ena bedekake mehakel)<br>04 ○ Coarse (Shekara)<br>05 ○ Very coarse (betam shekara)<br>06 ○ Do not know
3.27. On a scale from 1 to 10, how would you rate the soil quality of %S3Q15%?<br>I 1= VERY POOR SOIL; 10= EXCELLENT SOIL QUALITY<br>V1 S3Q27<=10	NUMERIC: INTEGER S3Q27<br>_ _ _ _ _ _ _ _ _ _ _ _ _ _ _ _ _ _ _ _
3.28. Is %S3Q15% soil acidic?	SINGLE-SELECT S3Q28<br>01 ○ Yes, it is an acidic soil<br>02 ○ No, it is not an acidic soil<br>03 ○ Do not know

SECTION 4: FIELD DESCRIPTION

4.1. LIST ALL FIELDS ON %S3Q15%	LIST S4Q1<br>..

SECTION 4: FIELD DESCRIPTION

Roster: 4.2. FIELD

generated by list question S4Q1 S4Q2

4.3. What is the FIELD ID of %rostertitle%?	NUMERIC: INTEGER S4Q3<br>_ _ _ _ _ _ _ _ _ _ _ _ _ _ _ _ _ _ _ _
4.4. What is the current state of %rostertitle%?	SINGLE-SELECT S4Q4<br>01 ○ Cultivated<br>02 ○ Pasture<br>03 ○ Fallow<br>04 ○ Forest<br>05 ○ Land Prepared for belg season<br>06 ○ Home/Homestead<br>07 ○ Other specify
4.5. What is the crop planted on %rostertitle% during the current agricultural season?<br>E S4Q4==1 | S4Q4==6	TEXT S4Q5<br>..
4.6. When did you plant the seeds for %rostertitle%?<br>E S4Q4==1| S4Q4==6	SINGLE-SELECT S4Q6<br>01 ○ September<br>02 ○ October<br>03 ○ November<br>04 ○ December<br>05 ○ January<br>06 ○ February<br>07 ○ March<br>08 ○ April<br>09 ○ May<br>10 ○ June

11 July
12 August
13 Pagume

4.7. Are you the primary decision maker regarding timing of cropping activities on this %rostertitle%?	SINGLE-SELECT S4Q7<br>01 Yes<br>02 No
4.8. What is the main water source of %rostertitle%?<br>E S4Q4==1 | S4Q4==6	SINGLE-SELECT S4Q8<br>01 Irrigation<br>02 Rain-Fed<br>03 Swamp / Wetland
4.9. Is %rostertitle% prevented from erosion?	SINGLE-SELECT S4Q9<br>01 Yes<br>02 No
4.10. What is the common way of preventing erosion on %rostertitle%?<br>E S4Q9==1 | S4Q4==6	SINGLE-SELECT S4Q10<br>01 Terracing<br>02 Water catchments<br>03 Afforestation<br>04 Plough along the line<br>05 Other (specify)
4.10b. Other:<br>E S4Q10==5	TEXT S4Q10b<br>..
4.15. How many times was %rostertitle% tilled in this agricultural season?<br>E S4Q4==1| S4Q4==6	NUMERIC: INTEGER S4Q15<br>_ _ _ _ _ _ _ _ _ _ _ _ _ _ _ _ _ _ _ _
4.16. Was chemical fertilizer used on %rostertitle% during this agricultural season?<br>E S4Q4==1| S4Q4==6	SINGLE-SELECT S4Q16<br>01 Yes<br>02 No
4.17. Did you use any UREA on %rostertitle% in this agricultural season?<br>E S4Q16==1	SINGLE-SELECT S4Q17<br>01 Yes<br>02 No
4.18. What was the quantity of UREA used on %rostertitle% in this agricultural season in kg?<br>E S4Q16 == 1 && S4Q17 == 1	NUMERIC: DECIMAL S4Q18<br>_ _ _ _ _ _ _ _ _ _ _ _ _ _ _ _ _ _ _ _
4.19. Did you use any DAP on %rostertitle% in this agricultural season?<br>E S4Q16== 1	SINGLE-SELECT S4Q19<br>01 Yes<br>02 No
4.20. What was the quantity of DAP used on %rostertitle% in this agricultural season in kg?<br>E S4Q16 == 1 && S4Q19 == 1	NUMERIC: DECIMAL S4Q20<br>_ _ _ _ _ _ _ _ _ _ _ _ _ _ _ _ _ _ _ _
4.21. Did you use any NPS on %rostertitle% in this agricultural season?<br>E S4Q16 == 1	SINGLE-SELECT S4Q21<br>01 Yes<br>02 No
4.22. What was the quantity of NPS used on %rostertitle% in this agricultural season in kg?	NUMERIC: DECIMAL S4Q22<br>_ _ _ _ _ _ _ _ _ _ _ _ _ _ _ _ _ _ _ _

E S4Q16 == 1 && S4Q21 == 1	
4.23. Did you use any other chemical fertilizers (other than UREA,DAP and NPS) on %rostertitle% in this agricultural season?<br>E S4Q16==1	SINGLE-SELECT S4Q23<br>01 ○ Yes<br>02 ○ No
4.24. What was (in kg) the quantity of other chemical fertilizers used on %rostertitle% in this agricultural season?<br>E S4Q16 == 1 && S4Q23==1	NUMERIC: DECIMAL S4Q24<br>_ _ _ _ _ _ _ _ _ _ _ _ _ _ _ _ _ _ _ _
4.25. Was organic fertilizer used on this %rostertitle% during this agricultural season?<br>E S4Q4==1	SINGLE-SELECT S4Q25<br>01 ○ Yes<br>02 ○ No
4.26. Did you use any manure on %rostertitle% in this agricultural season?<br>E S4Q25==1	SINGLE-SELECT S4Q26<br>01 ○ Yes<br>02 ○ No
4.26b. Did you leave any cows on %rostertitle%?<br>E S4Q26==1	SINGLE-SELECT S4Q26a<br>01 ○ Yes<br>02 ○ No
4.27. How many cow did you leave on %rostertitle% in this agricultural season?<br>E S4Q26a==1	NUMERIC: INTEGER S4Q27<br>_ _ _ _ _ _ _ _ _ _ _ _ _ _ _ _ _ _ _ _
4.28. For how many days per week did you leave cows on %rostertitle% in this agricultural season?<br>E S4Q27>0<br>V1 S4Q28<8	NUMERIC: INTEGER S4Q28<br>_ _ _ _ _ _ _ _ _ _ _ _ _ _ _ _ _ _ _ _
4.29. Did you use any compost on %rostertitle% in this agricultural season?<br>E S4Q25==1	SINGLE-SELECT S4Q29<br>01 ○ Yes<br>02 ○ No
4.30. What was the quantity of compost used on %rostertitle% in this agricultural season in kg?<br>I Enumerator:if they told you in traditional measurement ask them to guess it in KG<br>E S4Q29 == 1	NUMERIC: DECIMAL S4Q30<br>_ _ _ _ _ _ _ _ _ _ _ _ _ _ _ _ _ _ _ _
4.31. Did you use any other organic fertilizer on %rostertitle% in this agricultural season?<br>E S4Q25 ==1	SINGLE-SELECT S4Q31<br>01 ○ Yes<br>02 ○ No
4.32. What was the quantity used in this agricultural season in kg?<br>E S4Q25==1 && S4Q31 == 1	NUMERIC: DECIMAL S4Q32<br>_ _ _ _ _ _ _ _ _ _ _ _ _ _ _ _ _ _ _ _

SECTION 5: INDIGENOUS SOIL FERTILITY PERCEPTION

5.2. According to you, what are the characteristics of a fertile soil?<br><br>I DO NOT READ AND ASK UNTIL INTERVIEWEE LISTS ALL ITS INDICATO RS	MULTI-SELECT: ORDERED S5Q2<br>01 ☐ Dark in color<br>02 ☐ High crop yields<br>03 ☐ High water retention capacity<br>04 ☐ Easy to work<br>05 ☐ Numerous Wet Worm Casts<br>06 ☐ Large green leaves<br>07 ☐ Have 'soil animals' present<br>08 ☐ Soil type<br>09 ☐ Other (specify)
5.2a. Other:<br><br>E S5Q2.Contains(9)	TEXT S5Q2a<br>..
5.3. According to you, what are the characteristics of an infertile soil?<br><br>I MORE THAN ONE ANSWER	MULTI-SELECT: ORDERED S5Q3<br>01 ☐ Difficult to work<br>02 ☐ Give low yields<br>03 ☐ Are pale or light colored<br>04 ☐ Have low moisture holding capacity<br>05 ☐ Other (specify)
5.3a. Other:<br><br>E S5Q3.Contains(5)	TEXT S5Q3a<br>..
5.4. According to you, what are the causes of soil fertility decline?<br><br>I MORE THAN ONE ANSWER	MULTI-SELECT: ORDERED S5Q4<br>01 ☐ Ownership of land<br>02 ☐ Labor access<br>03 ☐ Capital access<br>04 ☐ Climatic changes<br>06 ☐ Changes in prices of agricultural inputs and outputs<br>07 ☐ Changes in agricultural policies<br>09 ☐ Lack of fertilizers use<br>10 ☐ Lack of fertility management<br>11 ☐ Other (specify)
5.4a. Other:<br><br>E S5Q4.Contains(11)	TEXT S5Q4a<br>..
5.5. On a scale from 1 to 10, how would you rate the quality of soils in the community?<br><br>I 1= VERY POOR SOILS IN THE EA ; 10= EXCELLENT SOILS IN THE EA<br>V1 S5Q5<=10	NUMERIC: INTEGER S5Q5<br>_ _
5.6. During the last 10 years, how has soil fertility evolved in the community?<br><br>E S2Q3>1	SINGLE-SELECT S5Q6<br>01 ○ It is much worse<br>02 ○ It is worse<br>03 ○ It is similar<br>04 ○ It is better<br>05 ○ It is much better
5.7. How would you judge the soil quality of your plots compared to other plots in the community?	SINGLE-SELECT S5Q7<br>01 ○ Much worse than other plots<br>02 ○ Worse than other plot<br>03 ○ Similar to other plots<br>04 ○ Better than other plots<br>05 ○ Much better than other plors
5.10. Record time	DATE: CURRENT TIME S5Q10

STATIC TEXT

END OF INTERVIEWEE QUESTIONNAIRE AND GO TO PARCEL %S3Q15%

SECTION 6: ENUMERATOR PARCEL VISIT

6.1. Record time	DATE: CURRENT TIME S6Q1 ....
6.2. How many trees can you see on [PARCEL]?	SINGLE-SELECT S6Q2<br>00 ○ 0<br>01 ○ 1-3<br>02 ○ 3-5<br>03 ○ >5

SECTION 6: ENUMERATOR PARCEL VISIT

PARCEL AREA MEASUREMENT WITH GPS

STATIC TEXT

Step 1. Open the COMPASS Application and identify the NORTHERN side of [PARCEL]

STATIC TEXT

Step 2. Turn on the GARMIN Etrex 20

STATIC TEXT

Step 3. Select SATELLITE icon and wait for the GPS signal to be less than 5m

STATIC TEXT

Step 4. Go back with back button and select AREA CALCULATION

STATIC TEXT

Step 5. Now click on START and go clock wise (North-East-South-West-North) to delimitate the [PARCEL]

STATIC TEXT

Step 6. Come back to your starting point (NORTHERN side) and click CALCULATE

6.3. Record the area of [PARCEL] in square meters	NUMERIC: DECIMAL S6Q3 _

STATIC TEXT

Step 7. click on Save Track and save the file with the Sample Bag ID name

SECTION 6: ENUMERATOR PARCEL VISIT

PARCEL ELEVATION

6.4. Record time	DATE: CURRENT TIME S6Q4 ....

STATIC TEXT

GO TO LOWEST POINT OF PARCEL AND SELECT COMPASS ICON ON THE GARMIN GPS

6.4a. What is the MINIMUM elevation of [PARCEL]?

NUMERIC: INTEGER S6Q4a

_ _

STATIC TEXT

GO TO HIGHEST POINT OF PARCEL AND SELECT COMPASS ICON ON THE GARMIN GPS

6.4b. What is the MAXIMUM elevation of [PARCEL]?

V1 S6Q4b>=S6Q4a

M1 Altitude must be > to minimum elevation

NUMERIC: INTEGER S6Q4b

_ _

6.5. Record time

DATE: CURRENT TIME S6Q5

....

STATIC TEXT

TURN OFF THE GARMIN ETREX 20

SECTION 6: ENUMERATOR PARCEL VISIT

ENUMERATOR SOIL QUALITY ASSESSMENT

6.5b. Weather conditions at time of assessment

SINGLE-SELECT S6Q5b

01 ○ Clear / Sunny
02 ○ Mostly clear
03 ○ Partly cloudy
05 ○ Completely cloudy
06 ○ Rainy

6.6. What is the soil color of [PARCEL]?

I DO NOT CITE, FIND THE CLOSEST ANSWER

SINGLE-SELECT S6Q6

01 ○ Light Grey (Fezaza Geracha)
02 ○ Dark Grey (Tekore yale geracha)
03 ○ Black (Tikur)
04 ○ Grey-Brown (gerachama buni teyem)
05 ○ Light Brown (Fezaza buni)
06 ○ Moderate Brown (mekakelegna buni)
07 ○ Dark Brown (tiqure buni)
08 ○ Pale Orange (Fezaza bertekuanema)
09 ○ Yellow-Orange (Bicha bertukanama)
10 ○ Yellow-Brown (Bicha buni)
11 ○ Other (specify)

Other: Precise the color

I Translate the color in english

E S3Q16==11

TEXT S6Q6a

....

6.7. VISUAL-AID-PROTOCOL: In which "block" is the soil color of [PARCEL]?

I USE THE RELEVANT VISUAL AID PROTOCOL

SINGLE-SELECT S6Q7

01 ○ N
02 ○ 5YR
03 ○ 10YR

6.8. What is the soil color of [PARCEL]?

E S6Q7==1

SINGLE-SELECT S6Q8

01 ○ N8
02 ○ N6
03 ○ N4
04 ○ N2
05 ○ N1

6.9. What is the soil color of [PARCEL]?

E S6Q7==2

SINGLE-SELECT S6Q9

01 ○ 5YR 8/2
02 ○ 5YR 8/4
03 ○ 5YR 6/2
04 ○ 5YR 6/4
05 ○ 5YR 6/6
06 ○ 5YR 6/8
07 ○ 5YR 4/2
08 ○ 5YR 4/4
09 ○ 5YR 4/6
10 ○ 5YR 4/8
11 ○ 5YR 2/2
12 ○ 5YR 2/4
13 ○ 5YR 2/6

6.10. What is the soil color of [PARCEL]?

E S6Q7==3

SINGLE-SELECT S6Q10

01 ○ 10YR 8/2
02 ○ 10YR 8/4
03 ○ 10YR 8/6
04 ○ 10YR 8/8
05 ○ 10YR 6/2
06 ○ 10YR 6/4
07 ○ 10YR 6/6
08 ○ 10YR 6/8
09 ○ 10YR 4/2
10 ○ 10YR 4/4
11 ○ 10YR 4/6
12 ○ 10YR 2/2
13 ○ 10YR 2/4

6.11. What is the slope on this [PARCEL]?

SINGLE-SELECT S6Q11

01 ○ Flat / nearly flat (tefetafa/medama)
02 ○ Gentle / moderate slope (medama mekakelegna daget)
03 ○ Strong slope (daget)
04 ○ Steep slope(Akebet)

6.12. VISUAL-AID-PROTOCOL: What is the slope on this [PARCEL]?

I USE THE RELEVANT VISUAL AID PROTOCOL

SINGLE-SELECT S6Q12

01 ○ Picture 1
02 ○ Picture 2
03 ○ Picture 3
04 ○ Picture 4
05 ○ Picture 5
06 ○ Picture 6
07 ○ Picture 7

6.13. What is the % of rocks on this [PARCEL]?

SINGLE-SELECT S6Q13

01 ○ 0%
02 ○ 2%
03 ○ 5%
04 ○ 10%
05 ○ 20%
06 ○ 30%
07 ○ 50% and more

6.14. VISUAL-AID-PROTOCOL: What is the % of rocks on this [PARCEL]?

I USE THE RELEVANT VISUAL AID PROTOCOL

SINGLE-SELECT S6Q14

01 ○ Picture 1
02 ○ Picture 2
03 ○ Picture 3
04 ○ Picture 4
05 ○ Picture 5
06 ○ Picture 6

	07 ○ Picture 7

SECTION 6: ENUMERATOR PARCEL VISIT
COMPOSITE SOIL SAMPLES

6.15. Record time	DATE: CURRENT TIME S6Q15<br>..

STATIC TEXT

ESTIMATE CENTER OF PARCEL AND PUT A STICK OR STONE IN POINT A

6.16. Record GPS point of the CENTER sample point (Point A)	GPS S6Q16<br>_ _ _ _ _ _ _ _ _ _<br>N<br>_ _ _ _ _ _ _ _ _ _<br>W<br>_ _ _ _ _ _ _ _ _ _<br>A

STATIC TEXT

TAKE 2 SAMPLES AND PLACE THEM IN THE BUCKET

6.18 What is the distance from A to NORTH border?<br>I USE COMPASS + DISTANCE APPLICATIONS	NUMERIC: INTEGER S6Q18<br>_ _ _ _ _ _ _ _ _ _
VARIABLE<br>S6Q18 /2	LONG S6Q19

STATIC TEXT

Walk %S6Q19% meters from NORTH corner to CENTER

6.20. Record GPS point of the NORTH sample point (Point B)	GPS S6Q20<br>_ _ _ _ _ _ _ _ _ _<br>N<br>_ _ _ _ _ _ _ _ _ _<br>W<br>_ _ _ _ _ _ _ _ _ _<br>A
6.21. Take a picture of the soil in front of you<br>I HOLD THE TABLET FLAT, AT A 1M DISTANCE FROM SOIL	PICTURE S6Q21

STATIC TEXT

TAKE 2 SAMPLES AND PLACE THEM IN THE BUCKET

STATIC TEXT

GO TO THE CENTER OF THE FIELD (Point A)

6.22. What is the distance from A to the NORTH-EAST border?<br>I USE COMPASS + DISTANCE APPLICATIONS (RESET BEFORE STARTING)	NUMERIC: INTEGER S6Q22<br>_ _ _ _ _ _ _ _ _ _
VARIABLE<br>S6Q22 - ((S6Q22*75)/100)	LONG S6Q23

STATIC TEXT

Walk %S6Q23% meters from the NORTH-EAST corner to CENTER

6.24. Record GPS point of the NORTH-EAST sample point (Point C)	GPS S6Q24<br>N<br>W<br>A

STATIC TEXT

TAKE 2 SAMPLES AND PLACE THEM IN THE BUCKET

STATIC TEXT

GO TO THE CENTER OF THE FIELD (Point A)

6.26. What is the distance from A to the EAST border?<br>I USE COMPASS + DISTANCE APPLICATIONS (RESET BEFORE STARTING)	NUMERIC: INTEGER S6Q26
VARIABLE<br>S6Q26 / 2	LONG S6Q27

STATIC TEXT

Walk %S6Q27% meters from EAST border to CENTER

6.28. Record GPS point of the EAST sample point (Point D)	GPS S6Q28<br>N<br>W<br>A
6.29. Take a picture of the soil in front of you<br>I HOLD THE TABLET FLAT, AT A 1M DISTANCE FROM SOIL	PICTURE S6Q29

STATIC TEXT

TAKE 2 SAMPLES AND PLACE THEM IN THE BUCKET

STATIC TEXT

GO TO THE CENTER OF THE FIELD (Point A)

6.30. What is the distance from A to SOUTH-EAST border?<br>I USE COMPASS + DISTANCE APPLICATIONS (RESET BEFORE STARTING)	NUMERIC: INTEGER S6Q30
VARIABLE<br>S6Q30 - ((S6Q30*75)/100)	LONG S6Q31

STATIC TEXT

Walk %S6Q31% meters from SOUTH-EAST border to CENTER

6.32. Record GPS point of the SOUTH-EAST sample point (Point E)	GPS S6Q32<br>N

	W<br>A

STATIC TEXT

TAKE 2 SAMPLES AND PLACE THEM IN THE BUCKET

STATIC TEXT

GO TO THE CENTER OF THE FIELD (Point A)

6.34. What is the distance from A to the SOUTH border?<br>I USE COMPASS + DISTANCE APPLICATIONS (RESET BEFORE STARTING)	NUMERIC: INTEGER S6Q34
VARIABLE<br>S6Q34 / 2	LONG S6Q35

STATIC TEXT

Walk %S6Q35% meters from SOUTH border to CENTER

6.36. Record GPS point of the SOUTH sample point (Point F)	GPS S6Q36<br>N<br>W<br>A
6.37. Take a picture of the soil in front of you<br>I HOLD THE TABLET FLAT, AT A 1M DISTANCE FROM SOIL	PICTURE S6Q37

STATIC TEXT

TAKE 2 SAMPLES AND PLACE THEM IN THE BUCKET

STATIC TEXT

GO TO THE CENTER OF THE FIELD (Point A)

6.38. What is the distance from A to the SOUTH-WEST border?<br>I USE COMPASS + DISTANCE APPLICATIONS (RESET BEFORE STARTING)	NUMERIC: INTEGER S6Q38
VARIABLE<br>S6Q38 - ((S6Q38*75)/100)	LONG S6Q39

STATIC TEXT

Walk %S6Q39% meters from SOUTH-WEST border to CENTER

6.40. Record GPS point of the SOUTH-WEST sample point (Point G)	GPS S6Q40<br>N<br>W<br>A

STATIC TEXT

TAKE 2 SAMPLES AND PLACE THEM IN THE BUCKET

6.42. What is the distance from A to the WEST border?<br><br>I USE COMPASS + DISTANCE APPLICATIONS (RESET BEFORE STARTING)	NUMERIC: INTEGER S6Q42<br><br>_________________
VARIABLE<br>S6Q42 / 2	LONG S6Q43

STATIC TEXT

Walk %S6Q43% meters from WEST border to CENTER

6.44. Record GPS point of the WEST sample point (Point H)	GPS S6Q44<br><br>_________________<br>N<br>_________________<br>W<br>_________________<br>A
6.45. Take a picture of the soil in front of you<br><br>I HOLD THE TABLET FLAT, AT A 1M DISTANCE FROM SOIL	PICTURE S6Q45

STATIC TEXT

TAKE 2 SAMPLES AND PLACE THEM IN THE BUCKET

STATIC TEXT

GO TO THE CENTER OF THE FIELD (Point A)

6.46. What is the distance from A to the NORTH-WEST border?<br><br>I USE COMPASS + DISTANCE APPLICATIONS (RESET BEFORE STARTING)	NUMERIC: INTEGER S6Q46<br><br>_________________
VARIABLE<br>S6Q46 - ((S6Q46*75)/100)	LONG S6Q47

STATIC TEXT

Walk %S6Q47% meters from NORTH-WEST border to CENTER

6.48. Record GPS point of the NORTH-WEST sample point (Point I)	GPS S6Q48<br><br>_________________<br>N<br>_________________<br>W<br>_________________<br>A

STATIC TEXT

TAKE 2 SAMPLES AND PLACE THEM IN THE BUCKET

STATIC TEXT

MIX COMPOSITE SAMPLES

PUT THE CONTENT INTO A SAMPLING BAG AND ZIP IT

STATIC TEXT

CLEAN THE BUCKET AND AUGER TO AVOID CONTAMINATION BETWEEN SAMPLES

6.50. Scan the sample bag barcode	BARCODE	S6Q50
6.51. Record time	DATE: CURRENT TIME<br>..	S6Q51

STATIC TEXT

TURN OFF THE GPS ON TABLET

LEGEND

Legend and structure of information in this file

Name of section

Enabling condition for this section

Question title

Type of question, scope

Variable name

Answer options

SECTION 5: OTHER INCOME SOURCES

E `s4_other_sources_which.Contains(98)`

Duis aute irure dolor in reprehenderit in voluptate velit esse cillum dolore eu fugiat nulla pariatur?

I **This refers to family relations**
E `s3_time_other > 0`
V1 `s4_rel_leaders_which.Contains(98)`
M1 **Can not be itself**
V2 `(s3_time_other_breeding_advice <= (50 - s3_time_art_in-sem_advice)) ||s3_time_other_breeding_advice == 0`
M2 **This person is not in the list**
F `optioncode != s5_ignored_option_code`

MULTI-SELECT
SCOPE: PREFILLED

`s4_rel_leaders_other`

01 ☐ **Community animal health workers**
02 ☐ **Private**
03 ☐ **Government**
04 ☐ **Livestock keepers association**
05 ☐ **NGO**

And 5 other [13]

Additional information:
"I" — Question instruction
"E" — Enabling condition
"V1" — Validation condition №1
"M1" — Message for validation №1
"F" — Filter in Categorical questions

Link to full set in appendix

Breadcrumbs

Type or roster

Roster Title

CHAPTER 3 IDENTIFICATION /
Roster: **LEADER RELATION DETAILS**
generated by fixed list:

01 **Ward Livestock Officer**
02 **Village Livestock Officer**
99 **Other (specify)**

List items
