# Supplementary material for: Challenges and lessons for measuring soil metrics in household surveys
Source: Geoderma. 2020 Oct 1;375:114500. doi: 10.1016/j.geoderma.2020.114500 (PMC7386900; doi:10.1016/j.geoderma.2020.114500)

Figure S4. Average raw spectra for organic C. Samples were air-dried, grinded and scanned with the Tellspec device (900–1700 nm). Vertical lines and dashed areas indicate NIR wavelengths that are highly predictive of organic C content.


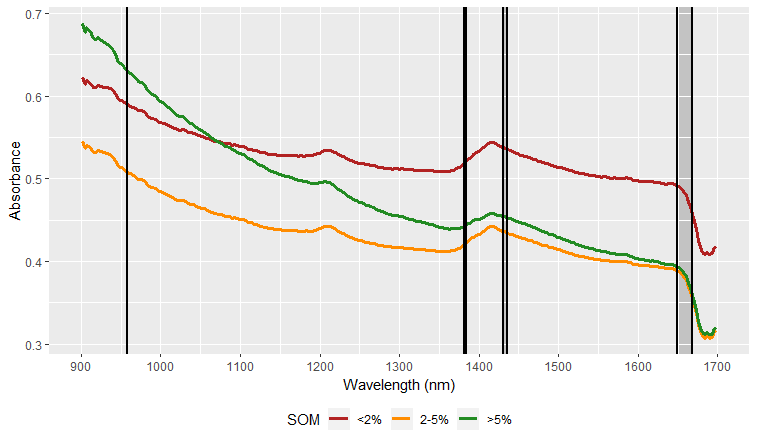

Supplement: Supplementary data 6 [file mmc6.zip › Appendix D. Average raw spectra for organic C]
